# Number of Seclusions in the Netherlands Higher in the 7 Years Since the End of a Nationwide Seclusion-Reduction Program

**DOI:** 10.3389/fpsyt.2021.778793

**Published:** 2021-12-03

**Authors:** Fleur J. Vruwink, André Wierdsma, Eric O. Noorthoorn, Henk L. I. Nijman, Cornelis L. Mulder

**Affiliations:** ^1^Mediant Geestelijke Gezondheidszorg, Enschede, Netherlands; ^2^Department of Psychiatry, Erasmus Medical Center, Epidemiological and Social Psychiatric Research Institute, Rotterdam, Netherlands; ^3^GGNet Geestelijke Gezondheidszorg, Warnsveld, Netherlands; ^4^Department of Social Sciences, Clinical Psychology, Behavioural Science Institute, Radboud University, Nijmegen, Netherlands

**Keywords:** seclusion, involuntary treatment, involuntary medication, involuntary hospitalization, psychiatry, nationwide program, seclusion reduction

## Abstract

**Introduction:** Between 2006 and 2012 the Dutch government funded a nationwide program for reducing the use of seclusion. Although an initial first trend study showed that the reported number of seclusions declined during the program, the objective of a 10% annual decrease was not met. We wished to establish whether the decline had continued after funding ended in 2012.

**Method:** Using quasi Poisson time series modeling, we retrospectively analyzed the nationally reported numbers of seclusion and involuntary medication between 1998 and 2019, i.e., before, during and after the end of the nationwide program, with and without correction for the number of involuntary admissions.

**Results:** With and without correction for the number of involuntary admissions, there were more seclusions in the seven years after the nationwide program than during the nationwide program. Although the reported number of involuntary medications also increased, the rate of increase was slower after the end of the nationwide program than before.

**Conclusions:** Rather than continuing to decrease after the end of the nationwide program, the number of seclusions rose. This may mean that interventions intended to reduce the use of seclusion within this program are not properly sustained in daily clinical care without an ongoing national program.

## Introduction

If other interventions in psychiatry fail, in many countries seclusion and restraint are often used as a last resort to manage disruptive and violent behaviors. Though both may prevent injury to the patient, others, or property, they also have negative side effects for patients and staff. These include not only negative feelings like anger, humiliation, anxiety and aggressive feelings, but also injury, disruption in the therapeutic relationship, and posttraumatic stress disorder (1–3).

Due to the relatively high seclusion rates in the Netherlands (4, 5), the Dutch government funded a nationwide program from 2006 to 2012 to reduce its use (6–10). Despite a 2% decrease in the use of seclusion between 2006 and 2009, the program did not meet its target of a 10% annual decrease. Meanwhile the increase of absolute number of involuntary medications did not change after the start of the program (the slopes of the increase before and after the start of the program were about the same) (11). The individual hospitals were free to choose an intervention to reduce the use of seclusion, leading to a wide range of new care methods to reduce the use of seclusion. These methods were for example structured risk assessment, feedback of data on coercive measures, deescalation training, trauma-informed care, increasing hospitality, but could also mean a changed building layout, like single-person bedrooms, comfort rooms, low-threshold access to nurses in the ward or at counters rather than in nurse stations (12, 13). In individual psychiatric hospitals the effects of the funding varied greatly, in some cases there were considerable reductions in the number and/or duration of seclusions; in other cases there were considerable increases (12). Overall, however the number of seclusions and their durations both decreased (12).

Internationally, there have been few studies of seclusion-reduction programs on such a large scale as an entire state and/or nation.

One such example covered the Seclusion and Restraint Reduction Program in Pennsylvania, where, after state-wide policy changes had led to a range of interventions, the state hospital system successfully reduced the use of seclusion and restraint nearly to zero between 1990 and 2000 (14). Until 2010 this successful program was still producing decreasing rates of seclusion (15). Elsewhere in the US, however, despite a national plan to reduce and ultimately eliminate the use of seclusion and restraint in mental health settings—including regulatory changes and support by important organizations — the rate of coercive measures in response to injurious assaults remained roughly constant at 438 adult psychiatric units in 317 hospitals between 2007 and 2013 (16). Välimäki et al. observed a similar pattern in Finland: despite the strong emphasis to decrease the use of coercive measures in psychiatric hospitals and a national action plan for 2009–2015 intended to increase awareness of the importance of reducing coercive measures, the actual reduction was small (17). Keski- Valkama et al. concluded that Finnish legislation had not been enough to reduce the use of seclusion and restraint over a 15-year period. It seemed that the prevailing treatment cultures had not really been challenged, and that the regional variations in Finland showed that the treatment traditions overpowered the law in different hospitals. As the authors indicate, the legislative changes would have yielded better results if they had been accompanied by national guidelines and a national educational program (18). None of these studies investigated the sustainability of continued reductions after the national programs ended.

There are several reasons why it is important to determine long term effects of such nationwide programs, for example the considerable investments of money and time and because little to nothing is known about their long-term effects.

The aim of this study was therefore to determine whether the nationwide decline in seclusion achieved during the reduction program had continued after funding ended. Even though it is not the primary focus of the program and our study, we also wished to determine whether there had been any changes in the national number of notifications of involuntary medications. We did this to ensure that a potential decrease in the use of seclusion did not lead to a concomitant rise in the number of involuntary medications (12).

## Methods

### Nationwide Program

Between 2006 and 2012, the Dutch government awarded grants to Dutch psychiatric hospitals that had specific plans for preventing the use of seclusions, and for carrying out any remaining seclusions more humanely. The most important criteria for qualifying for the grant, besides a seclusion reduction intervention, was that a psychiatric hospital had to monitor its results, and it had to match the sum it received (6–11).

In total, 73 (84%) of the 87 Dutch psychiatric hospitals with a permit for involuntary hospitalizations participated in the national program [Lists retrieved in an email conversation with L Willems, project manager of this nationwide program at the Dutch Mental Health Care Organization (GGZ Nederland), in February 2021 about the final reports of the funding of this nationwide program in 2012 (19)]. We assume that, by 2012, this number covered ~99% of the Dutch catchment area (12).

### Seclusion and Involuntary Medication

Seclusion was defined as locking a patient in a room designed for this purpose without opportunities to leave. Involuntary medication was defined as any medication administered (usually intramuscularly) against a patient's will. In the Netherlands, coercive interventions may only be used within an emergency measure (short term) or as part of a specifically elaborated involuntary treatment (long term) (4, 20). The start of either of these two ways of coercive measures has to be reported to the Dutch Health Care Inspectorate (DHCI), which published the annual numbers of notifications of seclusion and involuntary medication from 1998 until 2019. Thus, one notification could contain multiple episodes of the coercive measure in question (21). Other coercive measures, like mechanical restraint, are used little in the Netherlands (22, 23).

Under the mental health law that applied at the time, the Special Admissions Act [Wet Bijzondere Opnemingen in Psychiatrische Ziekenhuizen, Wet BOPZ (20)], seclusion and involuntary medication were permitted only with patients who have been admitted involuntarily. Involuntary hospitalization could be requested for inpatients and outpatients, if, as a consequence of their psychiatric illness, they caused danger to themselves or others, and also refused to consent with hospitalization or treatment. As the population at risk thus consists of patients who have been admitted involuntarily, we needed to know the number of involuntary hospitalizations. The number of requests for involuntary hospitalizations that had been processed from 1998 until 2009 was provided by the DHCI. The number of involuntary hospitalizations requested between 2010 and 2019 was obtained from the Dutch Council for the Judiciary (Raad voor de Rechtspraak) (24), which, unfortunately, collects only the number of processed requests for court-ordered involuntary hospitalizations, not the number of involuntary hospitalizations actually granted. However, the number of requests is a good indication of the real number of involuntary hospitalizations. For the 1998–2009 period the number of involuntary hospitalizations granted ranged between 94.7 and 97%; the mean number granted was 96.2%. We have no reason to assume that this trend was different in 2010–2019.

### Statistical Analyses

To model the time series data, we used a quasi Poisson Generalized Linear Model with a log link function and to account for autocorrelation we used the number of seclusions or involuntary medications in the previous year (25). To capture the effect of the nationwide program and overall trend “intervention period” and “year” (centered at 2006) were fixed covariates. As the intervention period was defined as running from 2006 through 2012, we defined 1998–2005 as being “before” the nationwide program, and 2013–2019 as being “after” it. To evaluate differences in developments before and after the intervention, we tested models that included an interaction term of period and year. Model selection was based on Wald-tests with alpha set at 5%.

To correct for changes in the number of involuntary hospitalizations, all models were replicated with the log of the number of involuntary hospitalizations per year as offset. Sensitivity analyses were conducted that limited the time series to 2006–2018. The purpose was to correct for possible registration bias caused by administrative start-up difficulties in the period to 2005, and also to correct for adaptations concerning legislative changes in 2019–2020 preparatory to the implementation of a new Dutch mental health law in 2020.

## Results

### Annual Numbers of Seclusion and Involuntary Medications

**Figure 1:**
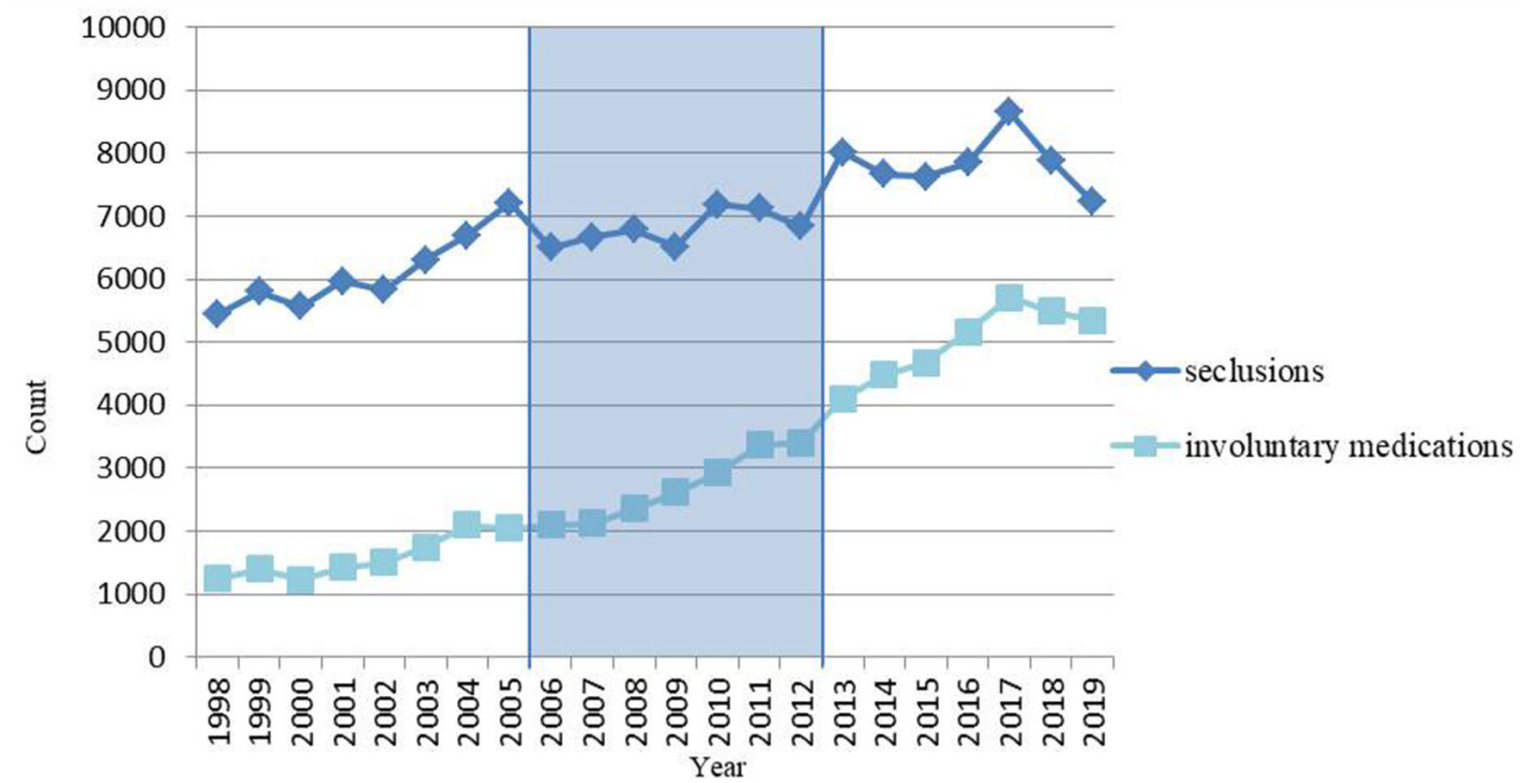
Number of seclusions and involuntary medications in the Netherlands per year, before (1998–2005), during (2006–2012) and after (2013–2019) a nationwide program to reduce the use of seclusion, in absolute numbers.

Between 1998 and 2005, the number of notifications of seclusions reported to the DHCI increased by 32.6%, representing an overall linear annual increase of 4.2%. Although the overall number of seclusions decreased (overall annual difference −0.78%) during the nationwide program, the number of seclusions increased by 5.7% in the seven years after the end of the program (overall annual difference 0.80%). The model presented in Table 1 suggests that the number of seclusions after the end of the nationwide program was indeed higher than during the program itself. However, the number of seclusions did not increase along a clear linear line (see Figure 1).

**Table 1:** Results of quasi Poisson time series models for the registered numbers of seclusion and involuntary medication in the Netherlands, both in absolute numbers and in numbers corrected for the number of involuntary hospitalizations (as used by off-set).

**Intervention**		**Absolute numbers**		**Corrected for number of involuntary hospitalizations**	
		**Estimate**	**95% CI**	**Estimate**	**95% CI**
Seclusions	Intercept	8.83		−1.059	
	Year^∙^	0.012	−0.0064 to 0.031	−0.012	−0.032 to 0.0094
	Number of interventions in previous year	−0.0000062	−0.000076 to 0.000064	0.000033	−0.000046 to 0.00011
	Period: before^#^	0.098	−0.0093 to 0.20	0.026	−0.095 to 0.15
	Period: after^#^	0.22	0.027 to 0.41	0.28	0.064 to 0.49
	Interaction year × period before^#^	0.029	−0.000018 to 0.057	−0.013	−0.045 to 0.020
	Interaction year × period after^#^	−0.015	−0.042 to 0.011	−0.027	−0.057 to 0.0031
Involuntary medication	Intercept	7.41		−2.38	
	Year^∙^	0.069	0.028 to 0.11	0.026	−0.014 to 0.067
	Number of interventions in previous year	0.00011	0.000037 to 0.00025	0.00020	0.000054 to 0.00034
	Period: before^#^	0.11	−0.053 to 0.27	0.011	−0.15 to 0.17
	Period: after^#^	0.50	0.27 to 0.72	0.60	0.38 to 0.82
	Interaction year* period before^#^	0.0041	−0.036 to 0.044	−0.023	−0.063 to 0.017
	Interaction year* period after^#^	−0.059	−0.093 to −0.024	−0.078	−0.11 to −0.044

The equation being: e^number of interventions^ = $e^{intercept+\;\beta_{1}\;*\;year\;number+\;\beta_{2}\;*\;number\;of\;intervention\;year\;before+\;\beta_{3}\;*\;period\;before+\;\beta_{4}period\;after+\;\beta_{5}\;*\;year\;number\;*\;period\;before+\;\beta_{6}\;*\;year\;number\;*\;period\;after}.$ “Intervention” is either seclusion or involuntary medication.

# *: Period “during” is the reference*.

∙ *: years are centered at 2006*.

What was remarkable in Figure 1 was a steep decrease in 2018 and 2019 to a level almost similar to that in 2010.

With regard to involuntary medications, the number reported to the DHCI between 1998 and 2005 increased by 64.1%, effectively a linear annual increase of 7.3%. The increase of the number of involuntary medications both during and after the nationwide program are comparable to the period before the program. But, in contrary to the numbers of seclusions, the annual number of involuntary medications was almost always greater than that in the previous year. Remarkably, however, there was also a steep decrease in 2018 and 2019.

The model (Table 1) shows not only that the number of involuntary medications after the end of the nationwide program was higher than during the program, but also that the increase over the period after the program was greater than that during the program.

The sensitivity analyses showed similar results. These analyses excluded the period before and the years 2018 and 2019. This means that these periods had no significant effect on the final estimates.

### Corrected Numbers

From 1998 until 2019, the number of involuntary hospitalizations increased (24). Since this is the population at risk, the analyses of the annual numbers of seclusions and involuntary medications were repeated and corrected for the number of the involuntary hospitalizations.

**Figure 2:**
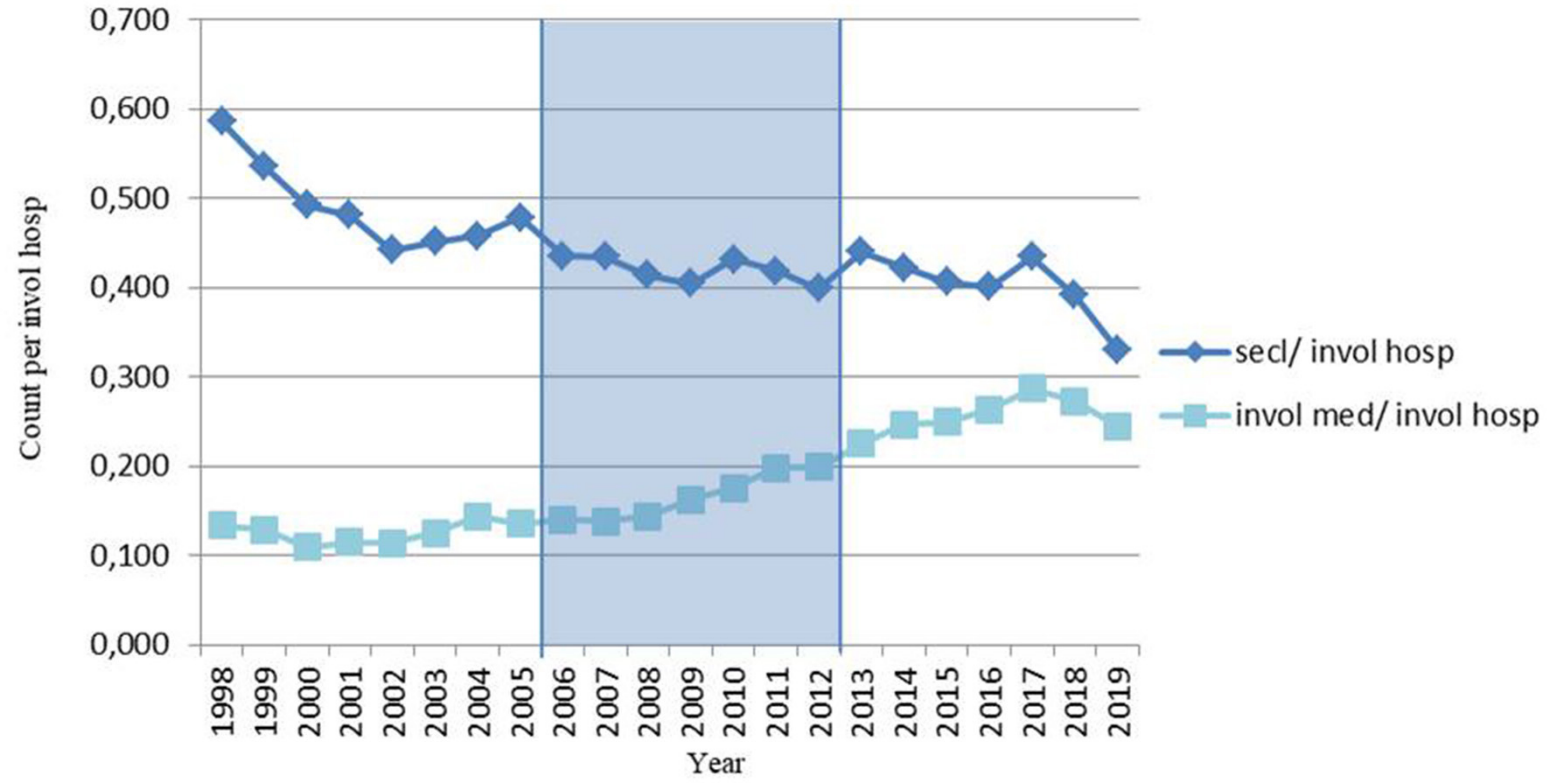
Number of seclusions and involuntary medications per involuntary hospitalization in the Netherlands per year, before (1998–2005), during (2006–2012) and after (2013–2019) a nationwide program to reduce the use of seclusion.

The number of seclusions reported to the DHCI using involuntary hospitalizations as an offset, decreased before, during and after the nationwide program with annual fluctuating percentages ranging between 2.5 and 2.9% per period (see Figure 2). And again, in line with the crude analyses in the time series model, the number of seclusions per involuntary hospitalization was higher after the nationwide program than during the program (see Table 1).

With regard to involuntary medications, the number reported to the DHCI per involuntary hospitalization increased by only 1.0% before the nationwide program (annual increase 0.14%). The annual increase was greater both during the program (5.7%) and after it (3.0%). In the time series model, the number of involuntary medications per involuntary hospitalization was greater after the program, although the slope was less steep (see Table 1).

For the estimates of the final time series models (see Table 1).

Again, sensitivity analyses showed similar results.

## Discussion

To our knowledge, few studies have examined national programs for reducing the use of seclusion, and no national scale or statewide study has examined the effects of such a program after it has ended. Ours is therefore the first to examine the longer-term sustainability of continued reductions after such a program. To determine whether a decrease in seclusions led to a concomitant increase in the use of involuntary medication, we also examined the use of involuntary medication. We found that, after funding ended, the number of seclusions and number of involuntary medications both increased.

### Seclusion

The decrease in the number of seclusions that took place during the nationwide program did not continue after the program ended: instead the numbers rose, an effect that remained even after correction for the increasing number of involuntary hospitalizations. This may mean that the effects of the nationwide program were not sustained in daily clinical care. Although one cannot predict what the number of seclusions would have been without any subsidy or funding, one may question whether the program justified the investment made in it.

The effects of the program may have been greater and better sustained if, in order to qualify for the government grant, the individual psychiatric hospitals had been obliged first to use only evidence-based methods to reduce the use of seclusions, and secondly to continue using them in daily clinical care after the end of the program. This would have ensured that the activities transcended the project itself by becoming embedded in normal daily practice.

We can only speculate about the lower number of seclusions in 2018 and 2019, which may be a new trend, with a cause that is as yet unknown, or otherwise an effect of ongoing efforts to reduce the use of seclusion and coercive measures. It may also be part of the varying pattern of seclusion numbers since 2012. As there was a similar decrease in the number of involuntary medications, it would be interesting to track these developments over the next few years.

### Involuntary Medication

During the 20 years under study, the registered number of involuntary medications in the Netherlands has continued to increase.

A partial explanation for the increase after 2008 may lie in the effect of certain changes in the Mental Health Act between 2004 and 2008, which were intended to broaden the options for involuntary treatment (22). For example, before these changes, the only legally allowable use of involuntary treatment was to prevent “serious danger” being caused by psychiatric illness. The deletion of the word “serious” from the new legal formulation broadened the options for involuntary treatment.

While it is conceivable that involuntary medication replaced seclusion, the higher number of seclusions in the last period make this unlikely.

Finally, we believe that the increased use of involuntary medications in the Netherlands reflects psychiatric health care workers' changing ideas and beliefs about treatment. It suggests a greater focus on using medication, whether voluntary or involuntary, to improve (in)patients' mental health rather than using seclusion to protect bodily integrity, which can be harmed by intramuscular injections (26). These changing ideas and beliefs have also been incorporated into the new Dutch mental health act: Act on Mandatory Mental Health Care [Wet verplichte geestelijke gezondheidszorg (27)].

### Sustainability

Although legal and cultural contexts differ between countries, it is interesting to compare our results with those of other national or statewide studies on reducing seclusion, and to see whether and how these results were sustained.

Pennsylvania's Seclusion and Restraint Reduction Program very successfully reduced the number of seclusions (14, 15). As this program started in 1990 and continued at least until 2010, we might assume that these practices have been sustained successfully in daily clinical mental healthcare. The ongoing focus on reducing the use of seclusion and restraint, because of continuing this program during decades, may have helped to sustain this effect. It would be interesting to see how the effects are sustained after this program ends.

The effect of the Dutch nationwide program we describe may be similar to that of the Finnish national plan for 2009–2015 (17), which, also, found non-linear changes in the use of coercive measures, with numbers going both up and down. However, Finland did show a small overall reduction in the use of coercive measure in the 20-year study period. To the best of our knowledge, no report has appeared on the continued sustainability of the Finnish national plan.

In the international literature on the sustainability of seclusion reduction programs, we found only one study—in the Netherlands—in which Mann-Poll et al. investigated the long-term effects of this nationwide program in three participating hospitals (28). Although, after the end of government funding, the three hospitals successfully reduced the use of seclusion, this study concluded that its effect soon disappeared once formal institutional awareness ended. During the funding period, the number of seclusions declined in all three hospitals. Afterwards, however, the situation varied. While the use of seclusion increased in the first hospital, and went up and down in the second, only the third hospital was able to maintain institutional awareness and to sustain lower seclusion rates.

Boumans et al. found that organizational context is very important. Although they found an initial reduction in the use of seclusion after the implementation of an innovation project in a psychiatric hospital, they also found during a later period of organizational turmoil that the staff's work engagement decreased and the use of seclusion increased again. This shows the vulnerability of innovations within an organizational context of continuous changes in mental healthcare (29).

Whitley et al. studied facilitating and barrier factors in the implementation of an innovation project. He found that leadership, organizational culture, training, staff and supervision played meaningful roles in determining the success or failure of its implementation, which was facilitated through strong leadership, an organizational culture that embraced innovation, effective training, and committed staff (30). These qualities, which even worked synergistically to effect implementation, sound similar to those applied in Pennsylvania (14), and are advocated in the much cited “Six Core Strategies for Reducing Seclusion and Restraint Use” by Huckshorn, which comprise: leadership toward organizational change; coercion data feedback; workforce development; coercion-prevention tools; consumer and family participation in all levels of the organization; and debriefing after every coercion incident (31). To attain sustainable results and prevent teams from falling back too easily into old routines, Mann-Poll et al. also advocated an ongoing developmental process of implementation (28).

Another initiative that might help to support the difficult task of sustaining a change in culture toward ongoing reductions in seclusion was proposed by Colton and Xiong, who developed a questionnaire intended to measure staff perceptions of organizational activities and staff attitudes toward the use of the interventions to reduce the use of seclusion (32).

### Limitations

Due to the retrospective observational design of our study, we cannot say whether the changes we observed in use of the coercive measures resulted directly from the government initiative.

The use of seclusion and involuntary medication may have been underreported to the DHCI (4), especially before 2006. A particular problem concerns the definition of involuntary medication, which leaves room for interpretation, as the boundaries between persuasion and coercion can be fluid. This may have led to underreporting on the use of involuntary medication.

As an extra check, we corrected the registered number of seclusions and involuntary medications for the number of processed involuntary hospitalizations. Although we believe this accurately represents the population at risk, it is not the actual number of involuntary hospitalizations, and comprises requests for short involuntary hospitalizations as well as those for longer ones. In addition, reasons for involuntary hospitalization might differ from reasons for seclusion and involuntary medication.

It is also possible that the increase in both the number of reported seclusions and involuntary medications was due to registration bias: in other words, that it was the product of better registration instead of an actual increase in the use of coercive measures. Although this may have been true for the years up until the start of the program, good registration of the number of seclusions and other involuntary measures then became mandatory as a condition for participation. For this reason, a form was developed on which detailed information could be entered on all the coercive measures applied, including seclusion and involuntary medication. This form, named Argus, was implemented nationwide, becoming mandatory from 2012 onwards for reports to the DHCI (33), and remaining so after the end of the program. As the number of reported seclusions varied greatly between 2009 and 2019, we assume that the numbers presented cannot be explained by better registration, especially since similar results were produced by our sensitivity analyses excluding the period before the start of the program.

### Conclusions

Rather than continuing the decrease after the end of the nationwide program, the number of seclusions rose. As this effect remained even after correction for the increasing number of involuntary hospitalizations, it may mean that interventions intended to reduce the use of seclusion within this program are not properly sustained in daily clinical care without an ongoing national program. To ensure that the effects of future seclusion-reduction programs or other national mental healthcare interventions are sustained after their subsidization ends, we recommend that such subsidies are granted only if these initiatives involve the implementation of evidence-based interventions in normal daily care. As advocated above, these initiatives should be accompanied by the Six Core Strategies for Reducing Seclusion and Restraint Use by Huckshorn, and an ongoing developmental process of implementation of the seclusion-reduction program in psychiatric hospitals.

## Data Availability Statement

The raw data supporting the conclusions of this article will be made available by the authors, without undue reservation.

## Ethics Statement

Ethical review and approval was not required for the study on human participants in accordance with the local legislation and institutional requirements. Written informed consent for participation was not required for this study in accordance with the national legislation and the institutional requirements.

## Author Contributions

FV, AW, EN, HN, and CM conceived and designed the study. FV collected and restructured the data. AW advised closely on the appropriate method of this study and performed the main analyses and critically revised the methods and results sections. FV wrote the first and following drafts, which EN and CM revised critically for important intellectual content. All authors approved the final version.

## Conflict of Interest

The authors declare that the research was conducted in the absence of any commercial or financial relationships that could be construed as a potential conflict of interest.

## Publisher's Note

All claims expressed in this article are solely those of the authors and do not necessarily represent those of their affiliated organizations, or those of the publisher, the editors and the reviewers. Any product that may be evaluated in this article, or claim that may be made by its manufacturer, is not guaranteed or endorsed by the publisher.

## Abbreviations

DHCI: Dutch Health Care Inspectorate.

## References

[1] Fisher WA. Restraint and seclusion: a review of the literature. Am J Psychiatry. (1994) 151:1584–91. 10.1176/ajp.151.11.1584 7943445

[2] Chieze M Hurst S Kaiser S Sentissi O. Effects of seclusion and restraint in adult psychiatry: a systematic review. Front Psychiatry. (2019) 10:491. 10.3389/fpsyt.2019.00491 31404294 PMC6673758

[3] Hawsawi T Power T Zugai J Jackson D. Nurses' and consumers' shared experiences of seclusion and restraint: a qualitative literature review. Int J Ment Health Nurs. (2020) 29:831–45. 10.1111/inm.12716 32198811

[4] Janssen WA Noorthoorn EO de Vries WJ Hutschemaekers GJ Lendemeijer HH Widdershoven GA. The use of seclusion in the Netherlands compared to countries in and outside Europe. Int J Law Psychiatry. (2008) 31:463–70. 10.1016/j.ijlp.2008.09.002 18954906

[5] Lepping P Masood B Flammer E Noorthoorn EO. Comparison of restraint data from four countries. Soc Psychiatry Psychiatr Epidemiol. (2016) 51:1301–9. 10.1007/s00127-016-1203-x 27147243

[6] College tarieven gezondheidszorg zorgauthoriteit,. Beleidsregel CA-65, Bijlage 6 bij Circulaire AVOS/masr/CARE/AWBZ/05/12c. (2005). Available online at: https://puc.overheid.nl/nza/doc/PUC_19411_22/1/ (accessed February 5, 2021).

[7] Nederlandse zorgautoriteit,. Beleidsregel CU-5011, Dwang en Drang in de Geestelijke Gezondheidszorg. (2009). Available online at: https://puc.overheid.nl/nza/doc/PUC_20562_22/1/ (accessed February 5, 2021).

[8] Nederlandse zorgautoriteit,. Beleidsregel CU-5024, Dwang en Drang in de Geestelijke Gezondheidszorg 2010. (2009). Available online at: https://puc.overheid.nl/nza/doc/PUC_20692_22/1/ (accessed February 5, 2021).

[9] Nederlandse zorgautoriteit,. Beleidsregel BR/CU-5037, Dwang en Drang in de Geestelijke Gezondheidszorg. (2010). Available online at: https://puc.overheid.nl/nza/doc/PUC_20815_22/ (accessed February 5, 2021).

[10] Nederlandse zorgautoriteit,. Beleidsregel BR/CU-5058, Dwang en Drang in de Geestelijke Gezondheidszorg. (2011). Available online at: https://puc.overheid.nl/nza/doc/PUC_20922_22/1/ (accessed February 5, 2021).

[11] Vruwink FJ Mulder CL Noorthoorn EO Uitenbroek D Nijman HL. The effects of a nationwide program to reduce seclusion in the Netherlands. BMC Psychiatry. (2012) 12:231. 10.1186/1471-244X-12-231 23249413 PMC3538066

[12] Noorthoorn EO Voskes Y Janssen WA Mulder CL van de Sande R Nijman HL. Seclusion reduction in dutch mental health care: did hospitals meet goals? Psychiatr Serv. (2016) 67:1321–27. 10.1176/appi.ps.201500414 27364814

[13] Voskes Y Theunissen J Widdershoven GA. Best Practices Rondom Dwangreductie in de Geestelijke Gezondheidszorg [translation: Best practices of reducing coercion in mental health care]. Amersfoort: GGZ Nederland (2012).

[14] Smith GM Davis RH Bixler EO Lin HM Altenor A Altenor RJ. Pennsylvania state hospital system's seclusion and restraint reduction program. Psychiatr Serv. (2005) 56:1115–22. 10.1176/appi.ps.56.9.1115 16148327

[15] Smith GM Ashbridge DM Davis RH Steinmetz W. Correlation between reduction of seclusion and restraint and assaults by patients in Pennsylvania's state hospitals. Psychiatr Serv. (2015) 66:303–9. 10.1176/appi.ps.201400185 25727119

[16] Staggs VS. Trends in use of seclusion and restraint in response to injurious assault in psychiatric units in U.S. Hospitals, 2007-2013. Psychiatr Serv. (2015) 66:1369–72. 10.1176/appi.ps.201400490 26174946

[17] Välimäki M Yang M Vahlberg T Lantta T Pekurinen V Anttila M. Trends in the use of coercive measures in Finnish psychiatric hospitals: a register analysis of the past two decades. BMC Psychiatry. (2019) 19:230. 10.1186/s12888-019-2200-x 31349787 PMC6660969

[18] Keski-Valkama A Sailas E Eronen M Koivisto AM Lönnqvist J Kaltiala-Heino R. A 15-year national follow-up: legislation is not enough to reduce the use of seclusion and restraint. Soc Psychiatry Psychiatr Epidemiol. (2007) 42:747–52. 10.1007/s00127-007-0219-7 17598058

[19] Van den Bos L Harmsen H. Slotrapportages GGZ Instellingen: Projectgelden terugdringen Dwang en Drang 2012[Translation: Final Reports Mental Health Care Institutions. Subsidies of Project Reducing Coercion 2012]. (2013). Available online at: https://docplayer.nl/11677364-Slotrapportages-ggz-instellingen-projectgelden-terugdringen-dwang-en-drang-2012.html (accessed November 8, 2018).

[20] Wet Bijzondere Opnemingen in Psychiatrische Ziekenhuizen (commonly referred to as Wet BOPZ) [translation: Special Admissions Act] (1992). Available online at: https://wetten.overheid.nl/BWBR0005700/2018-08-01 (accessed April 13, 2021).

[21] Janssen WA van de Sande R Noorthoorn EO Nijman HL Bowers L Mulder CL. Methodological issues in monitoring the use of coercive measures. Int J Law Psychiatry. (2011) 34:429–38. 10.1016/j.ijlp.2011.10.008 22079087

[22] Landeweer EGM Abma TA Berghmans RLP Dute JCJ Janssen WA Widdershoven GAM. Derde Evaluatie van de wet Bijzondere Opnemingen in Psychiatrische Ziekenhuizen, Deel 3: Dwangtoepassing Binnen de Instelling [translation: Third evaluation of the Special Admissions Act, part 3: coercive intervention within the institution]. Den Haag: Ministerie van Volksgezondheid, Welzijn en Sport. (2007).

[23] Steinert T Lepping P Bernhardsgrütter R Conca A Hatling T Janssen W. Incidence of seclusion and restraint in psychiatric hospitals: a literature review and survey of international trends. Soc Psychiatry Psychiatr Epidemiol. (2010) 45:889–97. 10.1007/s00127-009-0132-3 19727530

[24] Broer J Mooij CF Quak J Mulder CL. Stijging van BOPZ-maatregelen en dwangopnames in de ggz [Continuous increase in community treatment orders and compulsory admissions in the Netherlands, 2003-2017]. Ned Tijdschr Geneeskd. (2018) 162:D2454. 30358371

[25] Kedem B Fokianos K. Regression Models for Time Series Analysis. Hoboken, NJ: Wiley (2002).

[26] Verlinde AA Noorthoorn EO Snelleman W van den Berg H Snelleman-van der Plas M Lepping P. Seclusion and enforced medication in dealing with aggression: a prospective dynamic cohort study. Eur Psychiatry. (2017) 39:86–92. 10.1016/j.eurpsy.2016.08.002 27992811

[27] Wet Verplichte Geestelijke Gezondheidszorg [translation: Act on Mandatory Mental Health Care]. (2020). Available online at: https://wetten.overheid.nl/BWBR0040635/2021-07-01 (accessed July 20, 2021).

[28] Mann-Poll PS Noorthoorn EO Smit A Hutschemaekers GJM. Three pathways of seclusion reduction programs to sustainability: ten years follow up in psychiatry. Psychiatr Q. (2020) 91:819–34. 10.1007/s11126-020-09738-1 32279142

[29] Boumans CE Egger JI Bouts RA Hutschemaekers GJ. Seclusion and the importance of contextual factors: an innovation project revisited. Int J Law Psychiatry. (2015) 41:1–11. 10.1016/j.ijlp.2015.03.001 25846558

[30] Whitley R Gingerich S Lutz WJ Mueser KT. Implementing the illness management and recovery program in community mental health settings: facilitators and barriers. Psychiatr Serv. (2009) 60:202–9. 10.1176/ps.2009.60.2.202 19176414

[31] Huckshorn KA. Six Core Strategies for Reducing Seclusion and Restraint Use©. Available online at: https://www.nasmhpd.org/content/six-core-strategies-reduce-seclusion-and-restraint-use (accessed May 5, 2021).

[32] Colton D Xiong H. Reducing seclusion and restraint: questionnaire for organizational assessment. J Psychiatr Pract. (2010) 16:358–62. 10.1097/01.pra.0000388632.74899.86 20859114

[33] Schippers EI. Minister van volksgezondheid, welzijn en sport. Regeling van de Minister van Volksgezondheid, Welzijn en Sport van 15 december 2011, CZ-CGGZ-3093044, houdende wijziging van de Regeling kennisgeving en toepassing dwangbehandeling en middelen of maatregelen en registratie middelen of maatregelen Bopz [translation: Regulation of the Minister of Health, Welfare and Sport of 15 December 2011, CZ-CGGZ-3093044, amending the Regulation on notification and application of compulsory treatment and coercive measures and registration of coercive measures within the Special Admissions Act]. Staatscourant. (2011) 23432:1–5.

